# In utero exposure to electronic cigarette carriers alters craniofacial morphology

**DOI:** 10.1371/journal.pone.0327190

**Published:** 2025-06-30

**Authors:** Ethan Richlak, Logan Shope, Ethan Leonard, Leslie Sewell, Tyler Maykovich, Amr Mohi, Roy A. Miller, Matthew W. Gorr, Loren E. Wold, James J. Cray

**Affiliations:** 1 Nationwide Children’s Hospital Cleft Lip and Palate Center, Columbus, Ohio, United States of America; 2 University of Pittsburgh School of Medicine, Pittsburgh, Pennsylvania, United States of America; 3 The Ohio State University College of Dentistry, Columbus, Ohio, United States of America; 4 Department of Biomedical Education and Anatomy, The Ohio State University College of Medicine, Columbus, Ohio, United States of America; 5 Department of Cell Systems and Anatomy, University of Texas Health San Antonio, San Antonio Texas, United States of America; 6 Division of Cardiac Surgery, The Ohio State University College of Medicine, Columbus, Ohio, United States of America; 7 Divisions of Biosciences and Orthodontics, The Ohio State University College of Dentistry, Columbus, Ohio, United States of America; University Medical Center Hamburg-Eppendorf: Universitatsklinikum Hamburg-Eppendorf, GERMANY

## Abstract

**Objectives:**

Despite the popularity of electronic nicotine delivery systems (ENDS), there is currently a lack of regulation and consistency regarding the formulation of the e-liquids that undergo combustion in use. The two main constituents of most e-liquids are the humectants propylene glycol (PG) and glycerol (vegetable glycerin, VG). E-liquids consist of a ratio of these two components with PG utilized to increase the “throat hit” effect and VG used to increase visible vapor. As PG-based e-liquids are known to generate more carcinogenic carbonyls and increase the uptake of nicotine, many commercial products have moved toward a more VG-centric formulation to reduce potential harm. The purpose of this study was to test the hypothesis that a common VG-based formulation (30/70 PG/VG) would result in fewer negative effects on craniofacial growth compared to an evenly concentrated formulation (50/50 PG/VG) in the absence of nicotine.

**Materials and Methods:**

Adult breeder mice were utilized to generate *in utero* ENDS component exposed litters including free air exposure (control), 30/70 PG/VG, and 50/50 PG/VG groups. The resulting pups were assessed at postnatal day 14 for skull morphology.

**Results:**

Data demonstrate significant reductions in body weight, facial, and cranial dimensions, where there was a significant reduction in growth for the 30/70 PG/VG exposed group. There were no significant differences found between control and 50/50 PG/VG.

**Conclusions:**

These results suggest the overall movement to a more VG-centric ENDS formulation may not result in reduced profile for health concerns. Further, it suggests that PG/VG are not a harmless carrier and now popular nicotine-free ENDS formulation may not be considered safe for use in pregnant populations.

## Introduction

The emerging market of Electronic Nicotine Delivery Systems (ENDS) presents a substantial challenge to the public health community. ENDS products first appeared in the early 2000s and began to garner large market shares by the mid-2010s as engineering and sleek design furthered their popularity and use [1,2]. This is particularly true in younger populations (middle school, high school, and young adults) who, according to the ENDS industry, were not the anticipated target of use and marketing [1,3–11] as the products were instead meant to facilitate smoking and nicotine use cessation. There is now a new population of nicotine users owing to the popularity of ENDS. Public health concerns now include frequency of use, nicotine exposure levels, and gateway theories of use in a new cohort of tobacco-related products. There has been minimal response by regulatory agencies owing to a paucity of data and ENDS companies tying up government regulation in the court systems in the United States. Many questions remain but the long-term negative effects of ENDS products on addiction and disease are paramount. Despite this, the ENDS industry continues to expand and grow, creating a major setback to the advances that were made in public health when the use of tobacco products decreased [4].

Mixtures that are combusted when heated during ENDS use and ultimately inhaled are referred to as e-liquids. Despite many variations in the formulation of e-liquids, most of those commercially available include a carrier consisting of the humectants propylene glycol (PG) and glycerol, also known as vegetable glycerin (VG). PG and VG have been assessed for effects on health and new concerns exist [12–14]. In addition to humectants which allow for vaporization in ENDS, other major components include flavorants, acids (for pH balance), and often a variable concentration of nicotine. Much attention has been paid to nicotine and added flavorants, however, little focus has been given to the relative concentrations of PG and VG and if these humectants have health effects. Interestingly, product formulation is not a benign element in modeling the patterns of use or health effects, and this variability results in altered delivery and byproduct production of nicotine and carrier components. As carrier formulations vary from near pure propylene glycol, near pure vegetable glycerin, to mixed ratios in between, emerging research has been conducted to confirm delivery characteristics. One such study suggested that as propylene glycol ratios decrease, trace particulate matter and nicotine detection also decreases [15]. This data reinforces the need for additional *in vivo* models of physiological exposure to varied formulations of ENDS, which will allow for a better appreciation of safety standards and expected health effects with their use for multiple organ systems.

Data has consistently established that the use of traditional nicotine products during pregnancy can result in poor neonatal outcomes, and there is now a renewed focus on this relationship due to the increased use of ENDS [13,16–21]. According to the Centers for Disease Control and Prevention, approximately 3% of all newborns are afflicted with a birth defect. Birth defects of the head and neck are among the most common observed clinically [22,23]. The burden of these growth disorders is notable, as most require early and persistent surgical and therapeutic intervention to preserve life, vital organs, and restore form and function. Unfortunately, these interventions are rarely fully corrective [24–26]. Multiple craniofacial birth defects possess a genetic component with either a single causative gene, polygenic involvement, or a gene state that increases the risk of birth defects due to an insult or exposure [27–31]. In addition, numerous teratogens have been identified that instigate craniofacial birth defects in the absence of a genetic susceptibility [32–36]. Traditional tobacco products are known to behave as a teratogen by way of gene-environment interactions with respect to certain common craniofacial disorders including facial growth disorders, cleft lip and palate, and craniosynostosis [13,29,32–34,36–50]. Due to the heterogeneity in product formulation and use behaviors, a significant gap in knowledge exists as to how ENDS exposures might affect craniofacial development.

In the present study, we test two of the most common carrier formulation of ENDS, an even mixture of 50% PG, 50% VG, and a glycerol-centric formulation of 30% PG, 70% VG [51]. Emerging data suggests PG may be the more caustic of the two components, driving increased bioavailability of nicotine, flavorant byproducts, and adverse health effects [15,52]. Our hypothesis was thus that a PG/VG ratio of 50/50% will drive alterations in craniofacial development, where the reduction of PG in a PG/VG ratio of 30/70% will result in a reduced harm profile. To pursue this hypothesis, we are utilizing our model of *in utero* ENDS exposure and assessing resulting craniofacial morphology in the perinatal time period for the mouse.

## Materials and methods

Eight-week-old pregnant murine dams, C57BL6 (Jackson Laboratories 000664) were subjected to ENDS exposures during pregnancy under an approved IACUC protocol at The Ohio State University. Males were paired with females the day before the first exposure to ENDS aerosol, and females were exposed for 1 hour the first day, followed by 4 hours/day, 5 days/week until they gave birth. Exposures were conducted via the *Scireq inExpose* system (EMKA Technologies, Montreal QC, Canada). The real time exposure control system allows for regulation of nicotine concentration, puffs/minute, and time of exposure [21,53]. Exposures utilized were free (filtered) air control, 30/70 PG/VG, and 50/50 PG/VG.

Litters were allowed to age until sacrificed on postnatal day 14 to undergo cephalometric measurements. After sacrifice, the pups were weighed, and their skulls were fixed for Microcomputed Tomography (MicroCT) analyses. Methodology followed [54–62] using a Skyscan 1276 (Bruker Kartuizerseg 3B, 2550 Kontich, Belgium) scanner to garner 3D reconstructions for analysis. Using Analyze Pro software (Analyze Direct, Overland Park, KY), renderings were oriented and subject to cephalometric landmarking and measure of cranial, facial, and cranial base linear dimensions [37,54,56,57,60–64]. Data were compared by treatment (Free Air control, 30/70 PG/VG, 50/50 PG/VG). Further imaging used to illustrate morphological differences between exposures was modeled utilizing 3D Slicer [65] after segmentation of bony tissue. Anatomical overlays and 3D renderings were created from .tif files uploaded utilizing the SlicerMorph module.

This study was carried out in strict accordance with the recommendations in the Guide for the Care and Use of Laboratory Animals of the National Institutes of Health. The protocol was approved by the Ohio State University Institutional Animal Care and Use Committee (Protocol #2017A00000076-R1). Litters were housed with the dam for the duration of the experiment. All animal subjects were carried out to experimental endpoint of 14 days post-natal when euthanasia was conducted following American Veterinary Medical Association (AVMA) guidelines, specifically carbon dioxide overdose followed by vital organ removal. Care for enrolled animals was conducted under the guidance of Animal Care and Use Program at The Ohio State University including daily health and behavior checks. No *in vivo* methods or methods that induce stress or pain were conducted save breeding and aerosolization exposure to PG/VG, thus no anesthesia or analgesia were provided to animals to alleviate pain or suffering. No fetal or postnatal loss was observed and enrolled animals are enumerated in the following results section.

### Statistical analysis

Statistical analysis was conducted using SPSS 28.0 (IBM, Armonk, NY). Data were screened for normality and homogeneity of variance to assess whether analysis could proceed via parametric approach or if data were not normally distributed or did not exhibit equal variances a non-parametric alternative would be used. If assumptions were met, analyses followed using a one-way ANOVA with post-hoc Bonferroni analyses. If assumptions were violated, in the case of heterogeneity of variance a Welch’s correction to ANOVA was used. In the case where the assumption of normality was violated, a non-parametric approach was utilized using Kruskal Wallis with post-hoc Bonferroni analyses. No data transformations were conducted, and all variables were treated as continuous. Differences were considered significant if p < 0.05.

## Results

**Fig 1:**
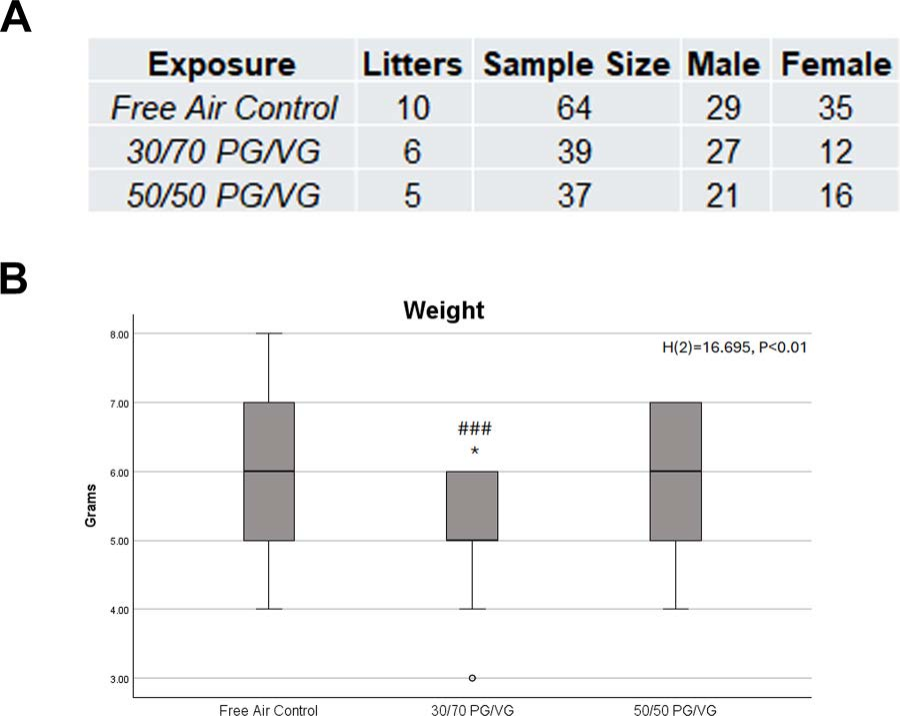
Sample size and weight measures. *A.* Sample Size denoted as litters and enumeration of postnatal 14-day pups. *B.* Kruskal Wallis test of independent samples revealed significant differences in weight by exposure. Post-Hoc Bonferroni analyses revealed the 30/70 PG/VG pups weighed significantly less than Free Air Control, p = 0.015, and 50/50 PG/VG, p < 0.001 respectively. * = p < 0.05 compared to control; ### = p < 0.001 compared to 50/50 PG/VG. Data represents weight in grams for the 3 exposure groups. Median is indicated by dark line, box represents 25th to 75th percentile, T-Bars represent 1.5 times the height of the box. Points outside the T Bars are indicated as outliers. Note the single outlier within the 30/70 PG/VG group.

21 litters were utilized for analyses, which resulted in 140 14-day postnatal pups,77 of which were male and 63 of which were female upon sex determination. All resulting pups were used for cephalometric analysis. The litters and resulting animals exposed to 30/70 PG/VG had significantly reduced weight at sacrifice compared to both Free Air Control and 50/50 PG/VG (Fig 1). It is worth noting that although there was a statistically significant reduction in weight, these weights were still within normal range for C57BL6 mice at 14 days of age [66]. Further data were screened for effect by sex or statistical interaction between exposure and sex. All analyses demonstrate that there are no differences by sex, suggesting that, at least at this age of the organism, sex is not influencing the response in growth (S1 Table).

**Table 1** included the description of cephalometric measures used [54,62,63,67] to determine any alteration in growth of the murine skull as a result of exposure groups studied here.

**Table 1:** Measures and descriptions.

Cephalometric Analysis (3D Rendered Crania)	
**PaNa**	Parietal point to Nasion
**Cw**	Cranial width; bilateral most lateral point of cranial vault
**FpOs**	Fronto-parietal suture to Occiput
**XPs**	Anterior sphenoid cranial base bone to Posterior occipital cranial base bone
**BS1BS2**	Width measures across bone present at Basion
**OpRh**	Opisthion to Rhinion
**Ms1Ms2**	Bilateral measure from where zygomatic arch articulates with the maxilla
**Mp1Mp2**	Bilateral measure from midpoint of zygoma
**Zp1Zp2**	Bilateral measure from where zygomatic arch articulates with the temporal bone
**PsRh**	Presphenoidal Ethmoidal Synchondrosis to Rhinion
**NaPs**	Nasion to Presphenoidal Ethmoidal Synchondrosis
**NaRh**	Nasion to Rhinion

**Fig 2:**
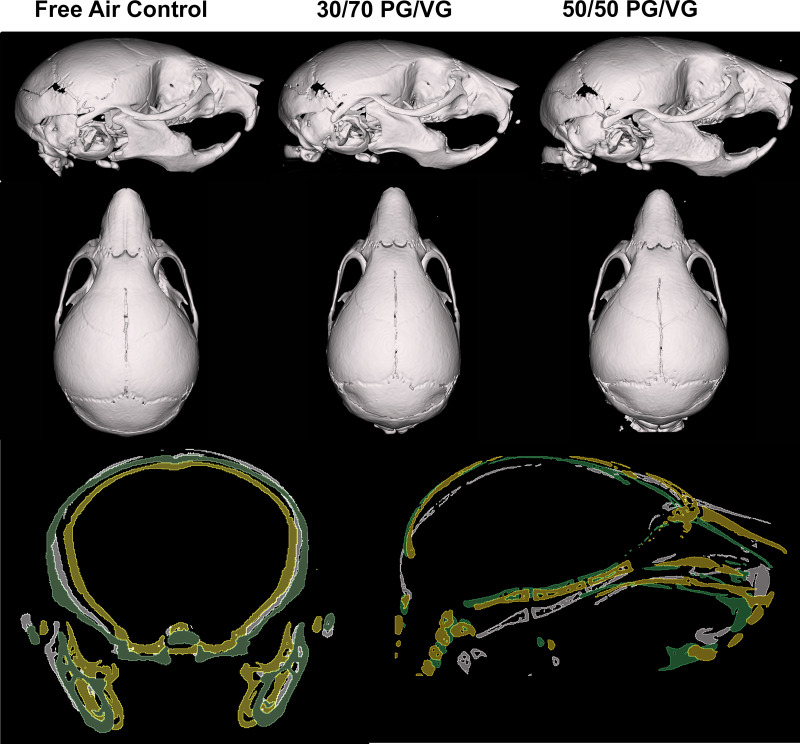
Change in skull form due to ENDS carrier exposure. *TOP:* Lateral 3D rendering of stereotypical skulls of *in utero* ENDS carrier exposures. MIDDLE: Superior View 3D rendering of stereotypical skulls of *in utero* ENDS carrier exposures. *BOTTOM:* Coronal (left) and sagittal overlays of stereotypical skulls of in utero ENDS carrier exposures. White/Grey = Control; Yellow = 30/70 PG/VG; Green = 50/50 PG/VG. Coronal overlay utilized sella turcica for anatomical registration. Sagittal overlay utilized C1 for anatomical registration.

**Fig 3:**
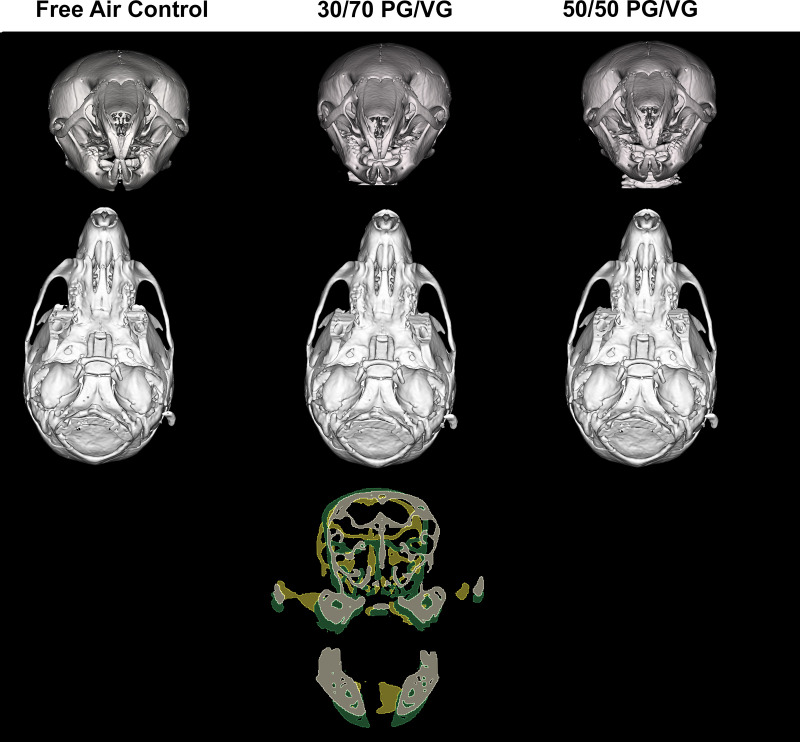
Change in skull form due to ENDS carrier exposure (Anterior and inferior view). *TOP:* Anterior 3D rendering of stereotypical skulls of *in utero* ENDS carrier exposures. MIDDLE: Inferior View 3D rendering of stereotypical skulls of *in utero* ENDS carrier exposures. *BOTTOM:* Coronal Facial overlays of stereotypical skulls of in utero ENDS carrier exposures. White/Grey = Control; Yellow = 30/70 PG/VG; Green = 50/50 PG/VG. Coronal Facial overlay utilized the rostral edge of the first molar for anatomical registration.

**Figs 2 and 3** demonstrate stereotypical skulls by exposure group in 3D rendering by various standardized views. The inclusive overlays included also emphasize alterations in several dimensions of the murine skull, specifically the face after exposures.

**Fig 4:**
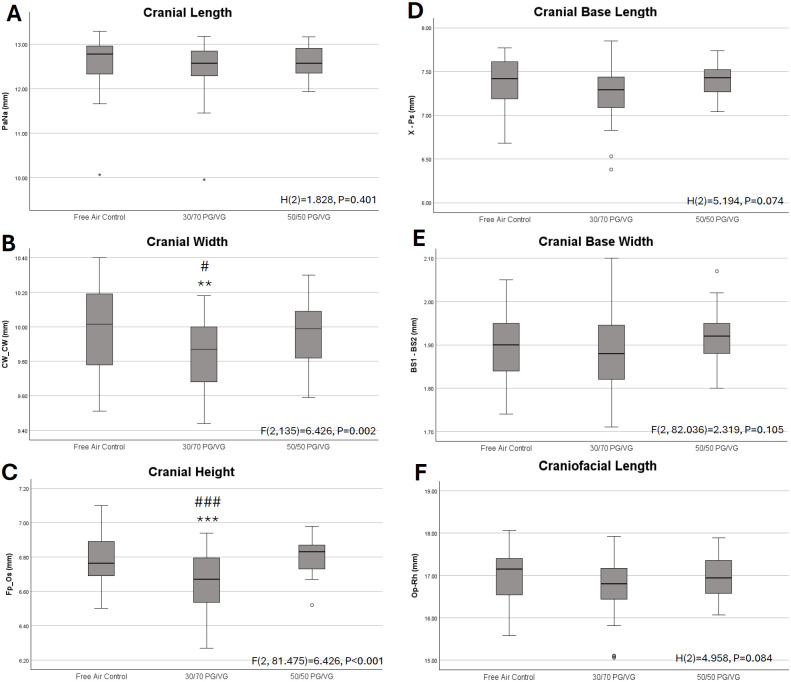
Cranial cephalometric measures. *A.* Kruskal Wallis test of independent samples for cranial length revealed no statistically significant differences by exposure. *B.* Analysis of Variance revealed significant differences in cranial width by exposure. Post-Hoc Bonferroni analyses revealed the 30/70 PG/VG pups had significantly reduced cranial width than Free Air Control, p = 0.002, and 50/50 PG/VG, p = 0.022 respectively. *C.* Analysis of Variance with Welch’s correction revealed significant differences in cranial height by exposure. Post-Hoc Bonferroni analyses revealed the 30/70 PG/VG pups had significantly reduced cranial height than Free Air Control, p < 0.001, and 50/50 PG/VG, p < 0.001 respectively. *D.* Kruskal Wallis test of independent samples for cranial base length revealed no statistically significant differences by exposure. *E.* Analysis of Variance with Welch’s correction for cranial base width revealed no statistically significant differences by exposure. *F.* Kruskal Wallis test of independent samples for craniofacial length revealed no statistically significant differences by exposure. ** = p < 0.01 and ***p < 0.001 compared to control; # = p < 0.05 and ### = p < 0.001 compared to 50/50 PG/VG. Boxplots are provided for additional context of growth variables. Data represented in millimeters for each growth variable for the 3 exposure groups. Median is indicated by dark line, box represents 25th to 75th percentile, T-Bars represent 1.5 times the height of the box. Points outside the T Bars are indicated as individual subjects statistically identified as outliers.

Cephalometric measures focused on the cranium revealed no significant alterations in the length dimension or in width as measured at the cranial base. However statistically significant differences were observed for cranial width and cranial height where the 30/70 PG/VG exposed pups were demonstrated to have reduced measures compared to Free Air Control and 50/50 PG/VG (**Fig 4**).

**Fig 5:**
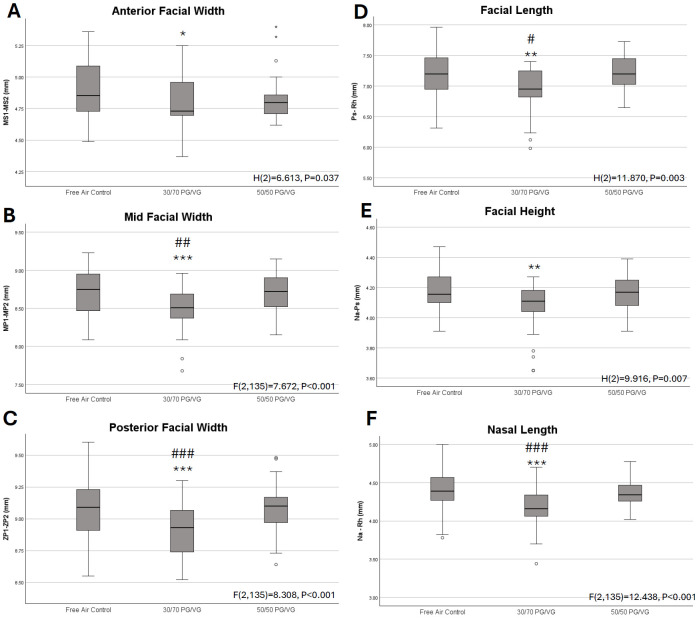
Facial cephalometric measures. *A.* Kruskal Wallis test of independent samples for anterior facial width revealed statistically significant differences by exposure. Post-Hoc Bonferroni Analyses revealed 30/70 PG/VG pups had significantly reduced width compared to Free Air Control, p = 0.045. *B.* Analysis of Variance revealed significant differences in midfacial width by exposure. Post-Hoc Bonferroni analyses revealed the 30/70 PG/VG pups had significantly reduced cranial width than Free Air Control, p < 0.001, and 50/50 PG/VG, p = 0.008 respectively. *C.* Analysis of Variance revealed significant differences in posterior facial width by exposure. Post-Hoc Bonferroni analyses revealed the 30/70 PG/VG pups had significantly reduced width than Free Air Control, p < 0.001, and 50/50 PG/VG, p = 0.004 respectively. *D.* Kruskal Wallis test of independent samples for facial length revealed statistically significant differences by exposure. Post-Hoc Bonferroni analyses revealed the 30/70 PG/VG pups had significantly reduced facial length than Free Air Control, p = 0.004, and 50/50 PG/VG, p = 0.016 respectively. *E.* Kruskal Wallis test of independent samples for facial height revealed statistically significant differences by exposure. Post-Hoc Bonferroni Analyses revealed 30/70 PG/VG pups had significantly reduced width compared to Free Air Control, p = 0.006. *F.* Analysis of Variance revealed significant differences in nasal length by exposure. Post-Hoc Bonferroni analyses revealed the 30/70 PG/VG pups had significantly reduced width than Free Air Control, p < 0.001, and 50/50 PG/VG, p < 0.001 respectively. * = p < 0.05, ** = p < 0.01, ***p < 0.001 compared to control; and # = p < 0.05, ## = p < 0.01, ### = p < 0.001 compared to 50/50 PG/VG. Boxplots are provided for additional context of growth variables. Data represented in millimeters for each growth variable for the 3 exposure groups. Median is indicated by dark line, box represents 25th to 75th percentile, T-Bars represent 1.5 times the height of the box. Points outside the T Bars are indicated as outliers.

Cephalometric measures focused on the facial skeleton revealed significant alterations in all analyzed dimensions. Statistically significant differences were observed for midfacial width, posterior facial width, facial length, and nasal length, where the 30/70 PG/VG exposed pups were demonstrated to have reduced measures compared to Free Air Control and 50/50 PG/VG. Further, there were statistically significant differences observed for anterior facial width and facial height when 30/70 PG/VG was compared to Free Air Control (**Fig 5**).

## Discussion

The purpose of this study was to examine how common formulations of e-liquids used in ENDS, in particular the ratio of two major humectants, propylene glycol and glycerol (vegetable glycerin), might alter craniofacial morphology. This is an important experimental approach to dissect the effects of carrier materials independent of the effects of nicotine or other ENDS additives which have received more focus. Here we utilized our established model [21,53] of murine exposure to ENDS, which has proven to be an appropriate method for studying *in utero* exposures and effects on developing systems [21,53,68,69]. Previous work has demonstrated that PG-centric formulations may result in greater adverse health effects than VG-centric formulations in a dose-dependent fashion [15]. This principle guided our hypothesis that exposure to a 30/70 PG/VG formulation *in utero* would result in reduced disruption to postnatal craniofacial growth compared to a 50/50 PG/VG formulation.

Overall our results were counter to the hypothesis and further question our current understanding of the effects of PG and VG on health as well as the safety of nicotine-free ENDS products which are becoming more popular. Here we demonstrate that cranial width and height measures were significantly altered within the 30/70 PG/VG cohort. This finding was reinforced when we interrogated the facial skeleton, in which decreased facial outgrowth was demonstrated for each measure when compared to the control groups, and in most cases, the 50/50 PG/VG group. Targeting of the facial skeleton is a trend we have observed in previous studies focused on nicotine exposures and are now seeing in nicotine-free ENDS carrier exposures, suggesting sensitivity in this anatomical area [21,55,57,59]. Some segregating decrease in postnatal weight was found in the 30/70 PG/VG cohort, though within normal range for this model [66]. Importantly, these results suggest that the overall shift to a more VG-centric ENDS formulation may not result in a reduced profile for health concerns. Further, our findings indicate that PG and VG are not inert chemical carriers in ENDS products. Thus, we conclude nicotine-free ENDS formulations should not be considered safe for use in pregnant populations. These findings are critical for informing the regulation of e-liquid formulations used in ENDS and to reduce the incidence of growth effects and disorders.

### Potential mechanisms of effect

While our data did establish a significant decrease in postnatal weight (albeit within normal range for our mice) between the 30/70 PG/VG and 50/50 PG/VG group, there is conflicting data within the literature surrounding the effects of ENDS exposure on somatic growth and weight. It is well-established that use of cigarettes during pregnancy can result in low postnatal weight [70]. The effects of ENDS is however an evolving scientific pursuit [71]. Multiple epidemiological studies have demonstrated an increased prevalence in low postnatal weight from ENDS exposure compared to those born to non-smokers [72,73]. Reduced postnatal weight has also been noted in preclinical studies as well; however, the identified studies used dams exposed to ENDS vapor which also included flavorants, which likely have their own health effects [74,75]. In contrast, there have been studies which demonstrated normal, or even increased, postnatal weight after ENDS exposure. One study compared the postnatal weight of babies born to mothers who only vaped, only smoked, smoked and vaped, and who have never vaped or smoked during the last trimester. These data demonstrated that mean postnatal weight of babies born to mothers who vaped was within 1 gram of the mean postnatal weight of babies born to mothers who neither vaped nor smoked [76]. This has also been demonstrated in preclinical models. Aslaner and colleagues found that murine pups exposed to 50:50 PG/VG without nicotine *in utero* did not display any significant disruptions to postnatal weight. Interestingly, they found that female mice exposed to 50:50 PG/VG with nicotine actually had a significantly higher body weight at adulthood [68]. Overall, the current literature suggests that weight outcomes between mothers who vape during pregnancy and mothers who do not vape during pregnancy are varied and require more study.

There is also a paucity of literature on the effects of nicotine-free PG and VG exposure *in utero* on overall body growth, and even less on craniofacial growth specifically, the focus of our study. Of the available work on the subject, the focus is mostly on inhalation toxicity and not organ and somatic development. There is however some existing literature that may provide clues into potential mechanisms that can account for the alterations of craniofacial and overall body growth. One study noted that a 50/50 PG/VG with nicotine ENDS exposure *in utero* resulted in the downregulation of multiple genes of the *Wnt* signaling pathway, including *Shh* [75]. The *Wnt* signaling pathway is known to be critical for neural crest cell migration and differentiation, as well as downstream osteogenesis and chondrogenesis [77]. Disruption in this pathway is a potential mechanism for the craniofacial growth disturbances observed in this study and fodder for future study. It is also worth noting that *Shh* is critical for guiding midface development [78], a cephalometric measurement that was consistently significantly impacted within our 30/70 PG/VG cohort. Further transcriptomic work is necessary to dissect the intricacies of the facial differences observed here in our model.

The toxicity of ENDS formulation is another growing area of concern. Kosmider et al. have demonstrated that the highest levels of the carcinogens formaldehyde and acetaldehyde were observed from ENDS vapors generated by PG-based formulations [79]. Additionally an investigation led by Moussa and colleagues investigated benzene (a known carcinogen when inhaled) emissions from ENDS with 6 different formulations, including 100% PG, 30/70% PG/VG, 100% VG, with the addition of nicotine.Their experiment concluded that the concentration of benzene in emissions actually increased as more VG was added into the formulation, suggesting VG would not be benign in carcinogenic exposures [80]. As many of these carcinogens have been noted to cause developmental toxicity [81,82] work will be necessary to determine if these additives or byproducts in emission could contribute to growth disturbances like those observed in our study.

While our work here focused on demonstrating that PG and VG alone can cause growth disturbances, the importance of continued studies of the effects of nicotine should not be understated. In ENDS exposures, the addition of nicotine has been demonstrated to increase the concentration of carcinogen emissions above PG/VG alone [71]. A more recent study found that *in utero* exposure to PG/VG both with and without nicotine changed the expression of multiple neuroinflammation genes significantly within the developing offspring [83]. There is noted literature highlighting the deleterious effects of maternal nicotine exposure on craniofacial development among humans and zebrafish [84–87]. What is clear is that nicotine exposure *in utero*, from any mechanism, continues to pose serious threats to numerous domains of growth and development and are a threat to public health.

### Future research

As highlighted in our study and by the review of the current landscape of literature on the adverse health effects of PG and VG, more work is needed to understand the cellular, molecular, and toxicological mechanisms of growth disruption. Future studies likely need to leverage both *in vivo* and *in vitro* tools to uncover the mechanisms by which PG and VG drive growth disruptions. In fact *in vitro* tools may be particularly amenable to a more broad scale approach to discover cellular mechanisms of effect, as what is clear is the variability in the carrier e-liquid for properties such as pH, viscosity, and differences in combustion products and concentrations of particulate matter is great, and likely influences critical cellular activities [15].

### Limitations

We have several limitations to highlight concerning these inclusive data and approaches. Anatomical differences, even when significant, in a preclinical murine model are historically difficult to appreciate and moreover the translation of this information from the murine model to the human is difficult. To contextualize the magnitude of effect of our results, deficiencies in growth for our significantly altered craniofacial variables span a 2–5% reduction in dimensional growth for this model. In the field of growth and development the question of catch-up growth is a salient one [88–92]. Further our data represents a single postnatal timepoint, and therefore we can only highlight this as a limitation and speculate as to what the consequences are of continued deficiency in facial outgrowth or the very real-world approach of watching and waiting for catchup growth, as would be encountered by those engaging in orthodontic or surgical interventions. A further limitation in design is that although we note no segregating difference in litter size by exposure or postnatal loss of pups, it is possible fetal resorption may have occurred, even in our control group, which was not appreciated.

This study, as designed, was targeted to address whether decreasing PG would prove less caustic than a carrier with more PG. In contrast our data suggest the 30/70 PG/VG exposure has more negative effects on craniofacial outgrowth and overall body growth. Importantly our data supports the paradigm that components found within ENDS products in isolation from nicotine may drive alteration in growth [93].

## Conclusions

Here we aimed to determine whether reducing propylene glycol in a mixed humectant carrier of propylene glycol and glycerol, PG/VG, would result in less effects on craniofacial outgrowth when exposed *in utero*. These results indicate that a 30/70% concentration of PG/VG resulted in more demonstrable alterations in craniofacial development compared to that of a 50/50% concentration of PG/VG. Moreover, the results indicate that devices and formulations that do not contain nicotine are not inherently benign. These findings indicate a significant need to further study ENDS components and formulations in isolation and mixture, as adverse effects on craniofacial growth were observed without the presence of nicotine or other common additives.

## Supporting information

S1 Table Sex as an independent variable. Sex was considered as an independent variable for each growth variable studied here within. Data was screened for normality and homogeneity of variance. If assumptions were met, we modeled a Two-Way ANOVA to determine if there were significant differences by sex or if there was a significant interaction term for sex by exposure for each growth variable of study. If normality was violated a Friedman’s test was carried out in a similar fashion using ranked data for those variables. For all growth variables studied, there were no significant differences by sex. Further, there were no significant interaction terms for sex by exposure. These data suggest no segregation by biological sex for response in growth by exposure modality.
(DOCX)
