# Supplementary material for: In utero exposure to electronic cigarette carriers alters craniofacial morphology
Source: PLoS One. 2025 Jun 30;20(6):e0327190. doi: 10.1371/journal.pone.0327190 (PMC12208421; doi:10.1371/journal.pone.0327190)
Supplement: S1 Table — Sex was considered as an independent variable for each growth variable studied here within. Data was screened for normality and homogeneity of variance. If assumptions were met, we modeled a Two-Way ANOVA to determine if there were significant differences by sex or if there was a significant interaction term for sex by exposure for each growth variable of study. If normality was violated a Friedman’s test was carried out in a similar fashion using ranked data for those variables. For all growth variables studied, there were no significant differences by sex. Further, there were no significant interaction terms for sex by exposure. These data suggest no segregation by biological sex for response in growth by exposure modality. (DOCX) [file pone.0327190.s001.docx]

**Supplemental Table 1: Sex as an Independent Variable.**

Sex was considered as an independent variable for each growth variable studied here within. Data was screened for normality and homogeneity of variance. If assumptions were met, we modeled a Two-Way to determine if there were significant differences by sex or if there was a significant interaction term for sex by exposure for each growth variable of study. If normality was violated a Friedman’s test was carried out in a similar fashion using ranked data for those variables. For all growth variables studied, there were no significant differences by sex. Further, there were no significant interaction terms for sex by exposure. These data suggest no segregation by biological sex for response in growth by exposure modality.

| Growth Variables | Sex as an Independent Variable | Sex x Growth Variable Interaction Term |
| --- | --- | --- |
| Weight | F=1.688, p=0.196 | F=1.008, p=0.295 |
| Cranial Length | F=1.201, p=0.275 | F=0.362, p=0.697 |
| Cranial Width | F=0.363, p=0.548 | F=0.713, p=0.492 |
| Cranial Height | F=0.022, p=0.883 | F=0.069, p=0.933 |
| Cranial Base Length | F=1.371, p=0.244 | F=0.002, p=0.998 |
| Cranial Base Width | F=0.014, p=0.905 | F=0.777, p=0.462 |
| Craniofacial Length | F=0.993, p=0.321 | F=0.161, p=0.851 |
| Anterior Facial Width | F=0.717, p=0.399 | F=0.338, p=0.714 |
| Mid Facial Width | F=0.333 p=0.565 | F=0.556, p=0.575 |
| Posterior Facial Width | F=0.042, p=0.838 | F=1.093, p=0.338 |
| Facial Length | F=0.101, p=0.751 | F=0.357, p=0.700 |
| Facial Height | F=1.666, p=0.199 | F=1.044, p=0.355 |
| Nasal Length | F=0.526, p=0.470 | F=0.452, p=0.638 |
